# Systematic review of caregiver burden, unmet needs and quality-of-life among informal caregivers of patients with pancreatic cancer

**DOI:** 10.1007/s00520-022-07468-7

**Published:** 2022-12-22

**Authors:** Eric Chong, Lisa Crowe, Keno Mentor, Sanjay Pandanaboyana, Linda Sharp

**Affiliations:** 1 Surgical and Translational Research Centre, Faculty of Medical and Health Sciences, University of Auckland, Auckland, New Zealand; 2 Population Health Sciences Institute, Newcastle University Centre for Cancer, Newcastle University, Newcastle, UK; 3 Department of Hepatobiliary, Pancreatic and Transplant Surgery, Freeman Hospital, Tyne and Wear, Newcastle Upon Tyne, UK

**Keywords:** Supportive care, Caregiver burden, Unmet needs, Quality-of-life, Pancreatic cancer

## Abstract

**Purpose:**

Informal caregivers play an important supportive care role for patients with cancer. This may be especially true for pancreatic cancer which is often diagnosed late, has a poor prognosis and is associated with a significant symptom burden. We systematically reviewed the evidence on caregiver burden, unmet needs and quality-of-life of informal caregivers to patients with pancreatic cancer.

**Method:**

PubMed, Medline, CINAHL and Embase databases were systematically searched on 31 August 2021. Qualitative and quantitative data on informal caregivers’ experiences were extracted and coded into themes of burden, unmet needs or quality-of-life with narrative synthesis of the data undertaken.

**Results:**

Nine studies (five qualitative, four quantitative), including 6023 informal caregivers, were included in the review. We categorised data into three key themes: caregiver burden, unmet needs and quality-of-life. Data on caregiver burden was organised into a single subtheme relating to symptom management as a source of burden. Data on unmet needs was organised into three subthemes need for: better clinical communication; support and briefings for caregivers; and help with navigating the health care system. Data on quality-of-life indicate large proportions of informal caregivers experience clinical levels of anxiety (33%) or depression (12%-32%). All five qualitative studies were graded as good quality; three quantitative studies were poor quality, and one was fair quality.

**Conclusion:**

High-quality pancreatic cancer care should consider the impacts of informal caregiving. Prospective longitudinal studies examining multiple dimensions of caregiver burden, needs, and quality-of-life would be valuable at informing supportive care cancer delivery to pancreatic cancer informal caregivers.

**Supplementary Information:**

The online version contains supplementary material available at 10.1007/s00520-022-07468-7.

## Introduction

Pancreatic cancer is a disease with high morbidity and a high mortality rate. The overall survival at 5-years is 6–10%, due in large part to late diagnosis which precludes curative resections [1, 2]. At diagnosis, only 10–15% of patients have localised disease and are potential candidates for curative resection [2, 3]. Even for patients who received resection, the overall 5-year survival rate is less than 25% because of early recurrence [1, 2]. Hence, the focus of treatment in the majority of the patients has been about achieving a balance between palliative chemotherapy and maintaining quality-of-life [4].

Informal caregivers — spouses/partners, other family member and/or close friends — serve an increasingly important role in the modern health system by supporting patients’ needs inside and outside the healthcare setting. In the context of cancer, informal caregivers may provide support during treatment, manage the patient’s medications, coordinate healthcare visits, and update friends and family, in addition to supporting patients with activities of daily living (e.g. feeding, bathing, and dressing patients) and instrumental activities of daily living (e.g. managing finance, cooking, and cleaning) [5, 6]. With a trend towards shorter hospital stays, the role of informal caregivers’ has expanded to include the monitoring and management of patients’ medication and symptoms [7]. Cancer caregivers are estimated to spend an average of 32.9 h a week providing care and with 72% of that duration spent on performing complex medical or nursing tasks [8].

The adverse effects of informal caregiving are increasingly appreciated in various diseases including cancer [9, 10]. In cancer, family members and friends are seldom prepared to be caregivers [11] and are thrust into the role at the time when they are coming to terms with the diagnosis. Disruptions to caregivers’ lives can be significant, and many suffer from a wide range of problems [12–14]. These effects can impact caregiver quality-of-life [15].

High-quality cancer care is now recognised as not limited to the delivery of appropriate treatment but also includes ensuring patients’ supportive care needs are met [16]. The scope of supportive care encompasses helping the patient *and their family* cope with cancer and its treatment across the illness trajectory and with death and bereavement [16]. The nature of the caregiver burden imposed by diseases varies according to the disease and its physical manifestations. The survival rate in pancreatic cancer is markedly lower than for other common cancers [17], and there has been little improvement over the past 30 years [18]. Most patients are diagnosed at an advanced stage, when curative treatment is impossible [19]. Patients tend to deteriorate rapidly and up to 90% have died within a year of diagnosis, demonstrating the aggressiveness of the condition [17]. All treatments and are associated with a significant side-effect burden, and patients’ quality-of-life and psychological wellbeing are notably worse compared to people without cancer and those with other forms of cancer [17]. It is therefore plausible that the impact of pancreatic cancer on informal caregivers is potentially distinct [18].

To inform the need for services and supports for informal caregivers of pancreatic cancer patients, it would be valuable to review the literature on the experiences of caregiving. This review aims to systematically identify and assess burden, unmet needs, and quality-of-life of informal caregivers of patients diagnosed with pancreatic cancer.

## Materials and methods

### Study selection

A systematic search was conducted using four databases: PubMed, Medline, Embase and CINAHL. The search syntax is presented in the Supplementary Table 1 (Appendix). In brief, this included terms for the disease, caregiving and burden, needs and quality-of-life. Databases were searched from inception to 31 August 2021. Both qualitative and quantitative studies were eligible, so no study design filters were applied in the searches. Reference lists of the included studies were screened to search for any additional studies not identified on the initial search and screening process.

Duplicate citations were removed. The remaining citations were screened by title and abstract by the first author (EC). Full texts of citations considered potentially eligible were obtained. Assessment of the full-text papers for eligibility was performed independently by two authors (EC and LC).

### Terminology and definitions

For the purposes of this review, caregiver burden included objective and subjective burden. Objective burden referred to tangible demands due to provision of care to the patient. Subjective burden referred to subjective experience of distress in the domains of health, psychological well-being, finances and social life, or due to the relationship between the caregiver and patient [20, 21]. Unmet needs were defined as needs which could be met with help from health care professionals, including allied health workers [22]. Quality-of-life was defined as the individual’s subjective perception of their physical, emotional, social and/or role wellbeing [21].

### Eligibility criteria

Inclusion criteria included studies that contained qualitative or quantitative data on the experiences of informal caregivers to patients with pancreatic cancer. Studies were included if they reported data on caregiver burden, needs of caregivers and/or caregiver quality-of-life, as defined above. To be eligible, studies had to report data collected from informal caregivers themselves. Exclusion criteria included: studies discussing caregiver experience indirectly (for example, data collected from patients or health professionals); studies not specific to pancreatic cancer; and studies that did not report separate caregiver experience for pancreatic cancer or that contained only data from a second-order analysis. Studies reporting interventions in caregivers were not eligible, as experiences and outcomes may be impacted by the intervention. Articles that were in a language other than English, case reports, editorials, opinion articles and review articles were also excluded. Conference abstracts were excluded as these generally lack sufficient detail for data extraction and synthesis and quality appraisal.

### Quality appraisal

Quality appraisal was undertaken independently by two authors (EC and LC). Any disagreements were reviewed with input from third author (LS). Qualitative studies were appraised using the Critical Appraisal Skill Programme (CASP) Qualitative Checklist [23]. The CASP Qualitative Checklist contains 11 questions based on three broader questions: ‘are the results of the study valid?’, ‘what are the results?’ and ‘will the results help locally?’ Points were assigned to responses to each question: ‘Yes’ = 2 points, ‘Somewhat’ = 1, ‘No’ = 0 and ‘Can’t tell’ = 0. The maximum total points for any study were 22. A priori, it was decided that study quality was considered good if the total score was 17–22 points, fair if 11–16 points and poor if 0–10 points.

Quantitative studies were assessed using the Methodological Index of Non-randomised Studies (MINORS) [24]. MINORS includes eight items for assessment of non-comparative studies (i.e. studies which do not include a control or comparator population) and twelve items for comparative studies. Points were assigned to possible responses to each question: ‘Adequately reported = 2’, ‘Reported but inadequate = 1’ and ‘Not reported = 0’. The maximum total points for comparative and non-comparative studies were 24 and 16 respectively. Quality of a study was considered good if the total points was 16–24, fair if 8–16 and poor if 0–8, irrespective of whether the study was comparative or not.

### Data extraction and synthesis

Data were extracted by two authors (EC and LC). Any disagreement in data extraction was resolved with input from a third author (LS). The extracted data included study aim, study design, country, duration, data collection method(s), number of informal caregivers included, caregiver age and gender, disease characteristics and data on caregiver experience associated with caregiver burden, unmet needs or quality-of-life.

EC (a medical practitioner) and LC (a qualitative researcher) conducted the data synthesis. Narrative synthesis of data was undertaken [25]; the limited number of eligible papers and heterogeneity in methods and outcomes precluded meta-synthesis. The aim of this study is to assess caregiver burden, unmet needs and quality-of-life. As such, these key areas formed our themes, using the definitions above, and data was coded using deductive thematic analysis [26]. In the event of any disagreement in definition due to overlap, a third reviewer (LS) was sought for categorisation.

From the papers, we took all reported quotes from caregivers and any caregiver-related statistics. Any similar quotes and statistics were coded into the themes. Quantitative data was restricted to quality-of-life only. When all data was grouped into the themes, two authors (EC and LC) went through the data to create the narrative synthesis of the current evidence on caregivers’ experience in the field of pancreatic cancer [25]. This analysis has enabled us to provide a summary of the current research available on caregiver burden in pancreatic cancer.

## Results

### Search results

**Fig. 1:**
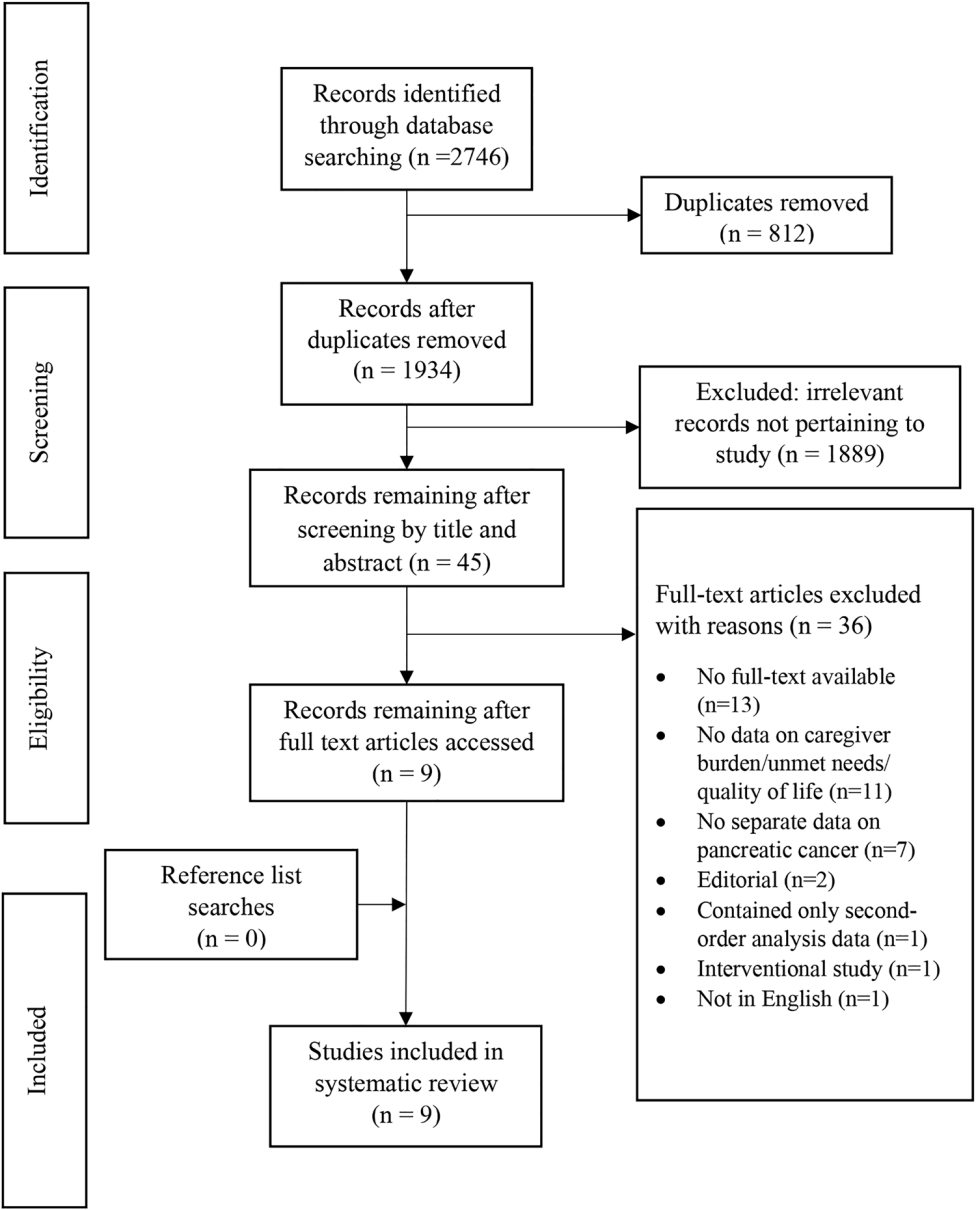
PRISMA flowchart of literature search

A PRISMA flowchart detailing the screening process is shown in Fig. 1. Briefly, the systematic search of databases returned 2746 citations. After removing duplicates and screening the remainder of articles by title and abstract, full texts of 45 citations were reviewed. Of these, 36 full texts were excluded. Manual searches of reference list of included studies did not return any further articles that met the inclusion criteria. Thus, nine studies were included in this review of which five were qualitative studies [27–31], and four were quantitative studies [5, 6, 32, 33].

### Descriptions of the studies

The nine studies were published between 2008 and 2021 (Table 1). A total of 6023 informal caregivers (inclusive of family members and first-degree relatives) were included. Excluding a large study which included 5574 informal caregivers [6], a total of 77 and 363 informal caregivers were included in the qualitative and quantitative studies respectively. Five studies (three qualitative, two quantitative) were conducted in the USA [5, 28, 29, 31, 33], three (two qualitative, one quantitative) in Australia [27, 30, 32] and one quantitative study in Denmark [6]. Data was collected through questionnaires alone in four studies [5, 32, 33], semi-structured interviews with individual participants in three studies [29–31], focus groups in two studies [27, 28] and questionnaire and semi-structured interview together in one study [29]. The Danish study conducted data linkage between the national cancer registry, civil registration system and the national prescription registry database [6]. Eight studies were cross-sectional studies [5, 27–33]. One study was a prospective cohort design [6].

**Table 1:** Study characteristics of eligible studies

Study (author and year), location	Study design	Study population	Data collection	Outcomes	Relevant findings/themes
Wong et al. (2019) [28], USA	Qualitative/cross -sectional study	25 informal caregivers	Semi-structured interview in focus group (photovoice method was used)	Psychological distress and coping with PC	Diagnosis of an unexpected, advanced cancer Changes in roles and identity Management of weight loss and GI symptoms Fear of the future
Sherman et al. (2014) [29], USA	Qualitative/cross-sectional study	7 informal caregivers	Semi-structured interview	Caregiver experience during diagnostic/treatment phase of illness	The caregiving context • History of illness: the sentinel event of significant symptoms • The crisis of diagnosis • The violation of assumptions about life and health • The circle of association • Contextual factors of family caregiving Primary stressors associated with family caregiving Secondary stressors association with family caregiving • Conduit of medical information
Gooden et al. (2013) [30], Australia	Qualitative/cross -sectional study	23 informal caregivers/family (including 14 bereaved caregivers)	Semi-structured interview (telephone or face-to-face)	PEI as an area of supportive care that requires an increased focus	Impact of symptoms on quality-of-life. Lack of information and dietary assessment Access to information about pancreatic EST Carer distress
Petrin et al. (2009) [31], USA	Qualitative/cross -sectional study	22 first-degree relatives (to 9 deceased and 11 alive patients with PC)	Semi-structured interview (telephone)	Experience of family members communicating and adjusting to first-degree relative’s diagnosis of PC, treatment, and subsequent death or survival	Reactions to initial diagnosis Coping with aftermaths of diagnosis Creating/maintaining support Family dynamics Considerations of future
Saunders et al. (2009) [27] Australia	Qualitative/cross -sectional study	11 participants (6 current and bereaved informal caregivers and 5 patients)	Semi-structured interview (telephone or face-to-face in group setting)	Consumer (i.e. patient or caregiver) thoughts and priorities in PC research	Early detection Clinical communication Public awareness Treatment options
Dengsø et al. (2021) [6], Denmark	Quantitative/retrospective prospective study	64,873 participants (5574 partners of PC patients, 59,099 partners of noncancer patients)	Data collected from DCR and CRS between 2000 and 2016	First incidence of depression, anxiety or insomnia	Higher first incidence of depression in partner of PC patients especially in first 2 years Risk factors for depression were female gender, older age, lower education levels and higher comorbidity Higher incidence of anxiety and insomnia in first year Higher incidence long-term insomnia
Janda et al. (2017) [32], Australia	Quantitative/cross-sectional study	84 informal caregivers	Questionnaires	Association between patient and carer wellbeing	Carer and patient level of distress and QoL were correlated
Sherman et al. (2015) [33], USA	Quantitative/cross-sectional study	64 informal caregivers	Questionnaires	Perceived social support, perceived health and depressive symptoms of informal caregivers of hospice patients with advanced pancreatic cancer	Family informal caregivers reported strong perceived social support, very good self-perceived health and low levels of depressive symptoms
Engebreston et al. (2015) [5], USA	Quantitative/cross-sectional study	213 informal caregivers (112 current informal caregivers, 102 bereaved informal caregivers)	Questionnaires	Perceptions about diagnosis and daily life with PC from patients and informal caregivers	Greater extent of negative emotional impact experienced by informal caregivers when compared with patients Significant differences in perception between informal caregivers and patients on the roles/tasks of informal caregivers

*CRS* Civil Registration System, *DCR* Danish cancer registry, *EST* endoscopic pancreatic sphincterotomy, *GI* gastrointestinal, *PC* pancreatic cancer, *PEI* pancreatic exocrine insufficiency, *QoL* quality-of-life, *USA* United States of America

Informal caregivers were predominantly spouses to patients with pancreatic cancer (98.6%, 6058/6142) [5, 6, 28, 33, 34]. The majority of caregivers were female (60.6%, 3748/6178). Most patients (74.7%, 4314/5772) cared for by the informal caregivers had advanced or metastatic pancreatic cancer.

### Quality appraisal

The five qualitative studies were critically appraised using the CASP qualitative study checklist (Table 2) [27–31]. All studies were considered good quality (17 to 22 points). From a maximum of 22 points, three studies scored 17 points [27–30] and two studies scored 18 points [31]. The lack of consideration for the relationship between researcher and participants [30, 31] and unsatisfactory data analysis [27–30] were the two most frequent reasons points were deducted during quality assessments.

**Table 2:** CASP Checklist for qualitative studies

Authors	CASP Checklist											
	Q1^a^	Q2^b^	Q3^c^	Q4^d^	Q5^e^	Q6^f^	Q7^g^	Q8^h^	Q9^i^	Q10^j^	Q11^k^	Total
Wong et al. (2019) [28]	SW	Y	SW	Y	Y	Y	N	Y	SW	Y	Y	17
Sherman et al. (2014) [29]	Y	Y	Y	Y	SW	Y	N	SW	SW	Y	Y	17
Gooden et al. (2013) [30]	Y	Y	Y	Y	Y	Y	N	Y	SW	Y	SW	18
Petrin et al. (2009) [31]	Y	Y	Y	Y	SW	Y	N	Y	SW	Y	Y	18
Saunder et al. (2009) [27]	Y	Y	Y	SW	SW	Y	N	Y	SW	Y	Y	17

*CASP* Critical Appraisal Skills Programme, *CT* cannot tell, *N* no, *SW* somewhat, *Y* yes

^a^Was there a clear statement of the aims of the research?

^b^Is qualitative methodology appropriate?

^c^Was the research design appropriate to address the aims of the research?

^d^Are the theoretical underpinnings, clear, consistent, and conceptually coherent?

^e^Was the recruitment strategy appropriate to the aims of the research issue?

^f^Was the data collected in a way that addressed the research issue?

^g^Has the relationship between researcher and participants been adequately considered?

^h^Have ethical issues been taken into consideration?

^i^Was the data analysis sufficiently rigorous?

^j^Is there a clear statement of findings?

^k^How valuable is the research?

The four quantitative studies were critically appraised using the MINORS tool (Table 3) [5, 6, 32, 33]. Three studies scored between 4 and 7 points and were graded as poor quality [5, 32, 33]. The cross-sectional design of the three studies meant they scored 0 points on items assessing duration of follow-up and loss of participants [5, 32, 33]. The one prospective study scored 14 points and was graded as fair quality [6]. All four studies scored 0 points for the item assessing prospective calculation of study size [5, 6, 32, 33].

**Table 3:** Methodological Index of Non-randomized Studies checklist for quantitative studies

Authors	MINORS questions												
	Q1^a^	Q2^b^	Q3^c^	Q4^d^	Q5^e^	Q6^f^	Q7^g^	Q8^h^	Q9^i^	Q10^j^	Q11^k^	Q12^l^	Total
Dengsø et al. (2021) [6]	2	2	0	2	2	2	0	0	1	2	0	1	14
Janda et al. (2017) [32]	2	1	2	2	0	0	0	0	0	0	0	0	8
Sherman et al (2015) [33]	2	0	2	2	0	0	0	0	0	0	0	0	7
Engebreston et al. (2015) [5]	2	1	0	1	0	0	0	0	0	0	0	0	4

*MINORS* Methodological index of nonrandomized studies, *NA* not applicable

^a^A clearly stated aim

^b^Inclusion of consecutive patients

^c^Data was collected according to established protocol

^d^Endpoint appropriate to the aim of the study

^e^Unbiased assessment of the study endpoint

^f^Follow-up period appropriate of the study

^g^Loss to follow-up less than 5%

^h^Prospective calculation of the study size

^i^An adequate control group was used?

^j^Studied and control groups were managed at the same time

^k^Baseline equivalence of studied and control groups

^l^Adequate statistical analyses were used

### Impact of informal caregiving in pancreatic cancer

Qualitative data of caregiver experiences were limited to describing caregiver burden and unmet needs. Supporting quotes illustrating caregiver experience are presented in Supplementary Table 2. Quantitative data on caregiver experience was limited to quality-of-life and is presented in Table 4.

**Table 4:** Quantitative evidence of caregiver’s quality-of-life

Mood symptoms	Quantitative evidence
Anxiety symptoms	HADS scores indicating clinical levels of anxiety were present in 39% of carers [30]. Feeling scared/anxious was reported by 12.2% of informal caregivers [5]. Significant association between patient and carer anxiety levels (*p* 0.027) [30]
	There was a significantly higher first acute use (1 prescription only) of anxiolytics in partners of PC patients than in comparators [6]
	Among patients and carers, accessing professional psychological help was significantly associated with subclinical or clinical HADS anxiety, and it was additionally associated with poorer QoL among patients [30]
Depression symptoms	Feeling sad or depressed was reported by 17.4% of informal caregivers [5], 15% of carers reported depression scores indicating clinical levels [30]
	The highest adjusted HR of first depression was seen the first year after diagnosis [6]
	Educational level, chronic morbidity and bereavement status were associated with an increased risk of first depression [6]
	Informal caregivers’ mean score on depressive symptoms, as measured by the CES-D, was low (2.8) but 32% of informal caregivers scored 4 or higher [31]
	The correlation between patients and carers levels of depression was high (correlation 0.53; *p* < 0.001) [30]

*CES-D* Centre for Epidemiological Studies Depression, *HADS* Hospital Anxiety and Depression Scale, *HR* hazard ratio, *QoL* quality-of-life

### Caregiver Burden

Three studies contained qualitative data illustrating caregiver burden [28–30]. No quantitative data has been reported on caregiver burden.

Qualitative data were organised into a single subtheme: managing patients’ symptoms as a source of burden. Informal caregivers were distressed by the management of patients’ symptoms which represented a subjective burden. Digestive symptoms (for example, loss of appetite [28, 30], weight loss [30], nausea [28], and constipation [28]) and side effects of treatment [28] were the sources of informal caregivers’ distress. Informal caregivers felt sad and ‘*helpless*’ when patients’ symptoms persisted despite the caregivers’ efforts to control them [30]. Some informal caregivers reacted with anger and frustration towards the situation which later led to guilt [30]. Guilt was also experienced by bereaved informal caregivers when symptoms were not adequately managed when patients were alive [30]. Apart from inability to control symptoms, informal caregivers also experienced guilt for pressuring patients to adhere to a dietary regimen or treatment plan [29, 30]. Guilt also arose when patients developed treatment side effects as caregivers viewed themselves as responsible in causing the side effects due to their role in supporting patients to receive treatment [29, 30]. Informal caregivers were also distressed by tension in the patient-caregiver relationship which arose when patients were unwilling/unable to continue a treatment plan [28].

### Unmet needs

Four studies reported qualitative data on unmet needs of informal caregivers [27–29, 31]. No quantitative data was available on unmet needs. The qualitative data were organised into three subthemes: need for better clinical communication, need for support and briefings for informal caregivers and need for help with navigating the health care system.

Two studies raised the need for better clinical communication [27, 29]. Informal caregivers described clinical communication at the time of diagnosis as insensitive [27]. They described the delivery of diagnosis as lacking empathy, as exemplified by phrases such as ‘*brutal*’ or ‘*not warm*’, and failing to treat the patient as a whole person [27]. Timing of delivery of diagnosis was also problematic with the possibility of cancer not mentioned ‘until after the surgery’ [29]. Unsatisfactory communication of medical decisions and rationales was reported by some informal caregivers [29]. Informal caregivers reported ineffective patient-clinician communication by mentioning, ‘*inconsistent advice*’ and ‘*information overload* [or] *underload*’ [29]. Informal caregivers suggested the development of ‘guidelines’ or ‘protocols’ to improve clinical communication in one study [27].

Three studies raised the need for support and briefings from health care professionals [28, 31] or a support system for informal caregivers [27, 31]. Informal caregivers felt there was a lack of interaction with medical providers or questions from medical professionals about their wellbeing despite their involvement in cancer care delivery [28]. They also reported feeling alone during the process of caregiving [31]. They reported they needed to ask clear questions to a provider [31] or have someone explain things to them [27], both of which represent unmet information needs.

Two studies raised the need for help with navigating health systems [28, 29]. Informal caregivers felt ‘*lost in the health care system*’ [28]. They suggested there was a need for ‘*a point person to serve as coordinator*’ [29].

### Quality-of-life

Four studies provided quantitative data on quality-of-life of informal caregivers [5, 6, 32, 33]. No studies provided qualitative data on quality-of-life of informal caregivers.

A study which surveyed 213 informal caregivers found that upon diagnosis of pancreatic cancer, 12.2% and 17.4% of informal caregivers reported feeling scared/anxious and sad/depressed [5]. In another study, including 64 informal caregivers to pancreatic cancer patients admitted to hospice care, depressive symptoms assessed using the short form of Centre for Epidemiological Studies Depression (CES-D) showed a low mean score of 2.8 (max score of 10) among informal caregivers [29]. However, 32% of the study population reported a score of 4 and above which is indicative of clinical depression [33]. A separate study including 84 informal caregivers who were recruited soon after the diagnosis of pancreatic cancer found that 14% and 17% of the informal caregivers had subclinical levels of anxiety and depression respectively, while 33% and 12% had clinical levels of anxiety and depression, based on the Hospital Anxiety and Depression Scale (HADS) [32].

Dengso et al. (2021) [6] used data-linkage methods and demonstrated a threefold higher adjusted hazard ratio for depression (HR 3.2 (95% CI: 2.9; 3.7)) in partners of pancreatic cancer patients in the first year after diagnosis when compared to partners of non-cancer patients, based on the proxy of first prescription for antidepressants or hospital admission for clinical depression. The same study showed that partners of pancreatic cancer patients had a higher chance of first acute use of anxiolytics when compared to partners of non-cancer patients [6].

Predictors of increased risk of first depression among pancreatic cancer caregivers were low education level, chronic morbidity, and bereavement [6]. Predictors of subclinical or clinical anxiety were a history of access to professional psychological help [32]. There was a correlation between anxiety or depression levels between patients and their informal caregivers [32].

## Discussion

The present systematic review, the first on caregivers of pancreatic cancer, has identified only nine studies, which reported qualitative or quantitative data on burden, unmet needs or quality-of-life, among informal caregivers to patients with pancreatic cancer. It identified the management of symptoms as a significant subjective burden among informal caregivers. It further identified three key unmet needs: the need for better clinical communication, the need of support and briefings for informal caregivers and the need for help with navigating health systems. It also found a high prevalence of depression and anxiety among informal caregivers.

Cancer patients tend to experience severe symptoms more frequently and to have a sharper decline in quality-of-life when compared to other chronic diseases [29]; this is particularly true in pancreatic cancer [2]. Healthcare policies and reforms in many countries now prioritize patients taking a more active role in (self-)managing their illness [29]; to effectively self-manage, patients need support, and that support often comes from a family member or friends [29]. Furthermore, health institutes’ policies also increasingly push for shorter length of hospital stay and more outpatient care thus placing greater responsibility on partners and families to supplement care received from professionals [29]. This evolution in the delivery of health care has led to the unintended sequelae of distress amongst caregiver when required to perform these caregiving tasks. It has been estimated that informal caregivers of cancer patients spend an estimated of 32.9 h a week on caregiving tasks, of which 72% involves performing complex medical or nursing tasks [35]. In the context of pancreatic cancer, the transition into caregiver role occurs very suddenly, and informal caregivers find themselves required to learn new medical information, coordinate appointment, manage medications, diet and nutrition, search for clinical trials and alternative treatments and prepare for medical emergencies within a short time span [28]. Given these trends and the aggressiveness of the disease course in pancreatic cancer in particular, modern health systems must allocate adequate resources to intervene and equip caregivers with basic medical or nursing competencies to cope with their caregiving task [36].

In our review, data on quality-of-life of informal caregivers was limited to their psychological wellbeing. The review showed large proportion of informal caregivers with clinical levels of anxiety (33%) and depression (12–32%). This level of psychological distress was similar to other studies on cancer caregivers [37]. Due to the lack of longitudinal studies, this review is not able to answer how pancreatic cancer caregivers’ quality-of-life changes across the trajectory of the illness; future studies on this would be of value. Predictors of psychological morbidity among informal caregivers were limited to sociodemographic and clinical characteristics including caregivers’ chronic morbidity, education status, bereavement, and history of access to professional psychological help [6, 32]. One study of patient-caregiver dyads showed correlations between anxiety and depression levels between patient and caregivers [32]. This suggests the possibility that interventions which support caregiver quality-of-life or psychological wellbeing may also yield benefits for patients (or vice versa).

Studies on cancer and non-cancer caregivers showed perceived burden as an important predictor of psychological morbidity [37, 38]. Different caregiving tasks, which constitute caregiver burden, impact caregiver distress to different extents. In one study on head and neck cancer, informal caregivers felt greater distress when assisting with caregiving tasks related to cancer-specific care (e.g. helping with medications) than those related to general supportive care (e.g. coordinating appointments) [39]. Feeling uncomfortable with cancer-specific care was predictive of informal caregivers’ anxiety and depression [39]. In this review, distress caused by managing pancreatic cancer symptoms featured prominently in the informal caregivers’ experiences. Future studies are needed to confirm the link between subjective burden of performing cancer-specific care and overall caregiver burden and their psychological quality-of-life in pancreatic cancer. Evidence that substantiates the proposed link would further justify the need to equip informal caregivers with necessary competencies to assist with cancer-specific care, in addition to providing psychological support to informal caregivers in need of such support.

Informal caregivers’ unmet needs identified in this review were the need for better clinical communication, need of support and briefings for caregivers and need for help with navigating the health system. Informal caregivers’ discontentment with clinical communication was largely directed at the delivery of diagnosis which they felt lacked empathy. In parallel with this, a survey of pancreatic cancer patients’ experience with delivery of news found two-third of patients felt their diagnosis was not given sensitively [40]. Ineffective patient-clinician communication, exemplified by contradictory, excess or inadequate information, was raised by a smaller subset of informal caregivers. This finding again echoes findings from the same patient survey wherein one-third of patients felt staff did not talk to them about their care and treatment in a way that they could understand [40]. Deficiencies in patient-clinician communication around cancer is an area that needs improvement; this is arguably especially important in a disease with such a poor prognosis, where the patient may decline very rapidly. Though tools such as guidelines and protocols have limitations, they can be useful adjuncts in supporting clinicians in navigating complexities of patient-clinical interactions when discussing cancer-related issues [41].

The second unmet need was the need for support and briefings from health care professionals. This again is related to communication with health professionals. As the focus of the health care system is to treat and care for the patient, informal caregivers felt neglected by the process of care delivery. This neglect has been studied by many researchers [42–44]. Despite the importance of informal caregiver to the modern health system, they report being seldom acknowledged, that health professional rarely show interest in them (and their health and ability to cope) and that health professionals spend insufficient time informing them about specific aspects of a disease such as symptoms and side effects [45]. Suboptimal interaction with informal caregivers may lead to caregiver distress and less competency in dealing with uncertainty and problem solving [42], thus potentially adversely impacting on the patient. This provides a rationale for seeking to find ways to better meet caregivers’ needs in this regard.

The final unmet need among pancreatic cancer informal caregivers is related to the need for help in navigating the health system. Cancer care delivery is complex, and patients may encounter multiple specialties including surgery, oncology, palliative, and occasionally emergency services across his/her disease course. Patient navigation programs could be one route to better meet caregiver (and patient) needs and improve access to the services [46].

Comparing our findings with those from past reviews suggests that there are some broad similarities between caregiving in pancreatic cancer and other cancers [14, 47]. For example, in a systematic review focused on caregiving in solid tumour patients, the management of symptoms also emerged as the foremost reported factor affecting burden [47]. In the review of Wang et al., on unmet needs in advanced cancer, the authors noted that several studies reported that caregivers had unmet needs in relation to illness and treatment information, which has some parallels with our findings, although ours are more specific [14]. Beyond this, direct comparisons between pancreatic cancer and other malignancies are difficult owing to the limited evidence base on caregiving in pancreatic cancer and differences in study methodologies.

Limitations of the review relate both to the review process and the nature of available evidence. We did not register the review protocol a priori with PROSPERO or another external database. A single author undertook the initial screening process of title and abstract. A ‘single screening’ approach is cost-effective and reduces workload [48], but risks missing studies which can impact the findings of the review [49]. Restricting inclusion to English-language studies also risked missing important evidence. Considering limitations in the evidence-base itself, quantitative data on caregiver burden and unmet needs is lacking and that on quality-of-life only covers a single dimension: psychological wellbeing. It is likely that caregivers’ role(s) across different phases of the illness trajectory evolve(s) with the changing tangible and intangible demands imposed on them. Prospective studies that track changes to caregivers’ experience (burden, needs and quality-of-life) across the trajectory of illness of pancreatic cancer are currently lacking. In addition, most data pertain to caregivers supporting patients with advanced or metastatic disease. Lastly, patient or caregiver characteristics, or other factors, that might be associated with greater burden, more needs and worse quality-of-life, and the interrelationships between different aspects of caregivers’ experience (burden, needs and quality-of-life) have largely not been examined. These limitations argue for studies with longitudinal design which use multi-method (i.e., qualitative and quantitative) approaches to characterise and understand multidimensional aspects (burden, needs and quality-of-life) of informal caregivers’ experience in pancreatic cancer across the illness trajectory. Such studies could also shed light on which caregivers are at greatest risk of poor experiences and the interrelationships between different aspects of caregiver experience.

## Conclusion

Informal caregivers of pancreatic cancer patients were burdened by management of patients’ symptoms. The unmet needs encountered were for better clinical communication, support and briefings for informal caregivers and help with navigating health systems. A large proportion of the informal caregivers experience anxiety and depression. Future longitudinal studies employing multi-method approaches are needed to better characterise and understand informal caregivers’ experiences.

## Supplementary Information

Below is the link to the electronic supplementary material.

Supplementary file1 (PDF 101 KB)

## Data Availability

All of the relevant data is contained within the article and supplementary material.

## Abbreviations

CASP: Critical Appraisal Skill Programme
CES-D: Centre for Epidemiological Studies Depression
MINORS: Methodological Index of Non-randomised Studies
