# Supplementary material for: Systematic review of caregiver burden, unmet needs and quality-of-life among informal caregivers of patients with pancreatic cancer
Source: Support Care Cancer. 2022 Dec 22;31(1):74. doi: 10.1007/s00520-022-07468-7 (PMC9771849; doi:10.1007/s00520-022-07468-7)
Supplement: Supplementary file 1 — Supplementary file1 (PDF 101 KB) [file 520_2022_7468_MOESM1_ESM.pdf]

<u>**APPENDIX**</u>

Supplementary Table 1

#	Key word	Possibilities
**1**	**Cancer**	**Pancreatic Neoplasms OR Pancreatic cancer OR Cancer of the pancreas OR Pancreatic adenocarcinoma**
**2**	**Quality of life**	**Anxiety OR Anxiety disorders OR Depression OR Psychological distress OR Quality of life**
**3**	**Unmet needs**	**Unmet need* OR needs assessment* OR perceived need* OR Support care need OR psycho* need* OR physical need* OR information* need* OR function* need***
**4**	**Burden**	**Burden OR Strain**
**5**	**Caregiver**	**Caregiver* OR Carer* OR Family OR relative* OR Family relations**

#6 = #1 and #5

#7 = #2 OR #3 OR #4

#6 AND #7

Supplementary Table 2 Supporting caregiver quotes on burden and unmet needs

Themes	Subthemes	Illustrative quotes
Caregiver burden	Managing symptoms as a source of burden	"I was trying to keep his weight going and it was just so difficult…I'd encourage him to eat and then he had bad stomachs and oh (crying)… and always burping." Carer/Female (Dyad) [28]<br><br>"I found this very confronting, her not wanting to eat. I (crying) try to force feed her. She gets upset, it makes it worse." Carer, Male (Dyad) [28]<br><br>Caregiver guilt experienced when "wanting to help a patient eat "or "get angry and frustrated" when the patient "doesn't have the energy to do things." It also came from "pressuring her to continue treatment" despite the symptom side effects and patient's feelings that "enough is enough." [27]<br><br>"He is trying to protect me and still wants to be in charge so that I am not as stressed but I want to do things for him because he is the one who is sick.'' [27]<br><br>"mentally draining and extremely frustrating" (Jason) [26]<br><br>Well, I am helpless. I can give her emotional support but I can't cure the cancer, I can't make her not nauseous, I can't make her go to the bathroom, I can't make her eat normally...so it's very frustrating because there's so many things out of my control (Jason). [26]<br><br>"We only got it [pancreatic enzyme supplement] about a week or 10 days out [before she died] and it would have been better if we'd had them a month earlier." Carer, Male (Bereaved). [28]
Unmet Needs	Need for better clinical communication	"I think guidelines would be helpful there, taking into account the sensitivity of the individual" [25]<br><br>"(The) ways that the doctors come out and tell you. There have to be some guidelines that they need to go by in a situation like this" [25]<br><br>"When the actual first diagnosis was given to us, we had an unfortunate experience as it was rather brutal. My wife was told to get her affairs in order. That's not treating the individual as a whole person. We just felt very badly let down" [25]<br><br>"I didn't feel that the manner of delivery was particularly warm. It's almost like some of the specialists are so experienced with what they're doing that they lose sight of the fact that for the person who's been diagnosed, it's the first time they've ever heard these words. Very often what's been told is there is actually nothing that can be done. Maybe there could be a set of words and communication could be improved a little bit?" [25]<br><br>"That conversation regarding pancreatic cancer is never going to be a nice one and we have to avoid shooting the messenger there. Maybe there could be some protocol that specialists follow a little more thoroughly. No-one is ever going to want to hear it so it's never going to be well received" [25]<br><br>"Anything we mentioned was virtually cast aside, almost contemptuously... The prognosis may be dismal, but my wife was entitled to a lot more than that. The human spirit demands more than that" [25]<br><br>'The word cancer was never mentioned until the doctor came to speak to us after the surgery, we were in total shock.'' [27]

		Family informal caregivers spoke of the …' "differing medical opinions,'' "inconsistent advice," or "information overload or underload.'[27]
	Need for support and briefings for caregivers	"Even when you go in and see Dr. X or any of the other providers, it's about them [the patients]. Nobody says how are you doing? (Gretchen)." [26]
		[T]here was no support system. A support group or any group or resources out there that would assist the family that can, you know, go through the process. Same way you feel like the only one going through it and have no provider that we can ask clear questions. (Offspring, living relative) [29]
	Need for help with navigating the health care system	"I think you reach a point where you can't read anymore and you can't search the internet anymore and you just need somebody to sit next to you and talk to you and explain things to you" [25].
		"lost in the health care system.'' [27]
		"Where do we begin? We need help!"[26]
		"with no point person to serve as coordinator.'' [27]
